# Association between *RsFT*, *RsFLC* and *RsCOL5* (*A&B*) expression and flowering regulation in Japanese wild radish

**DOI:** 10.1093/aobpla/plab039

**Published:** 2021-06-23

**Authors:** Qingxiang Han, Shota Sakaguchi, Tomomi Wakabayashi, Hiroaki Setoguchi

**Affiliations:** 1 College of Life Sciences, Zaozhuang University, Zaozhuang City, Shandong Province, 277160, China; 2 Graduate School of Human and Environmental Studies, Kyoto University, Kyoto, 606-8501, Japan

**Keywords:** Flowering time, natural selection, photoperiod, vernalization, wild radish

## Abstract

Flowering is an important step in the life cycle of plants and indicates adaptability to external climatic cues such as temperature and photoperiod. We investigated the expression patterns of core genes related to flowering-time regulation in Japanese wild radish (*Raphanus sativus* var. *raphanistroides*) with different vernalization requirements (obligate and facultative) and further identified climatic cues that may act as natural selective forces. Specifically, we analysed flowering-time variation under different cold and photoperiod treatments in Japanese wild radish collected from the Hokkaido (northern lineage) and Okinawa (southern lineage) islands, which experience contrasting climatic cues. The cultivation experiment verified the obligate and facultative vernalization requirements of the northern and southern wild radish accessions, respectively. The expression of major genes involved in flowering time indicated that *RsFLC* and *RsCOL*5 (*A*&*B*) may interact to regulate flowering time. Notably, floral initiation in the northern lineage was strongly correlated with *RsFLC* expression, whereas flowering in the southern linage was correlated with induction of *RsCOL5-A* expression, despite high *RsFLC* transcript levels. These results suggested that the northern accessions are more sensitive to prolonged cold exposure, whereas the southern accessions are more sensitive to photoperiod. These different mechanisms ultimately confer an optimal flowering time in natural populations in response to locally contrasting climatic cues. This study provides new insights into the variant mechanisms underlying floral pathways in Japanese wild radish from different geographic locations.

## Teaser

To identify climate cues that might act as natural selective forces shaping the genetic variation for flowering initiation, we conducted analyses of the flowering-time variation in response to cold treatment and day length in wild radish collected from Hokkaido and Okinawa islands with vernalization requirements. Our results collectively offer a hint that accessions with obligate vernalization requirements might exhibit a higher sensitivity to prolonged cold exposure, whereas in accessions with facultative vernalization requirements the photoperiod pathway predominates, and these mechanisms confer an optimal flowering time in natural populations, in response to their contrasting climate cues.

## Introduction

Flowering is the developmental turning point from the vegetative to reproductive phase. The induction of flowering is the most important stage regarding reproductive strategy and the allocation of limited resources (Komeda 2004). Variation in flowering time is a key feature of the life histories of flowering plants and determines crucial aspects of plant reproductive ecology. As such, there has been intense interest in determining the genetic architecture of this trait (Fitter and Fitter 2002; Schlenker and Roberts 2009; Li *et al.* 2010; Raman *et al.* 2016; Xiao *et al.* 2019). In *Arabidopsis thaliana*, for example, the precise timing of flowering transition is regulated by a complex hierarchical signalling network that integrates many environmental and endogenous stimuli (Huijser and Schmid 2011; Weber and Burow 2018).

Various factors, including photoperiod, temperature, plant age and gibberellic acid (GA) content, converge to regulate FLOWERING LOCUS T (*FT*) expression in flowering plants (Fornara *et al.* 2010; Cho *et al.* 2017). Among multiple seasonal cues, temperature plays an essential regulatory role, particularly in plants that require an extended cold period to initiate flowering, known as vernalization (Michaels and Amasino. 2000). A key component in the vernalization regulatory network in *Arabidopsis* is *FLOWERING LOCUS C* (*FLC*), a MADS-box transcription factor that quantitatively inhibits floral transition by encoding a *FT* transcription repressor (Michaels *et al.* 2003; Deng *et al.* 2011). *FLC* plays a critical role in monitoring seasonal temperature trends under fluctuating natural environments (Satake *et al.* 2013; Zheng *et al.* 2018). Vernalization stably represses *FLC* expression in response to prolonged cold exposure, and thus accelerates flowering, whereas *FRIGIDA* (*FRI*) promotes high *FLC* expression and thereby prevents flowering (Michaels and Amasino 2001; Searle *et al.* 2006). Allelic variations in *FRI* and *FLC* account for much of the natural variation in *Arabidopsis* flowering time (Shindo *et al.* 2005). For example, the winter-annual ecotypes contain dominant *FRI* and *FLC* alleles and require vernalization for rapid flowering (Michaels and Amasino 2000), whereas many summer-annual and rapid-cycling ecotypes flower quickly without vernalization via disruption of the *FLC* regulatory sequences (Michaels *et al.* 2003) and absence of an active *FRI* allele resulting in low *FLC* expression (Johanson *et al.* 2000).

In addition to temperature, photoperiod (day length) is a major environmental factor that affects the timing of floral transition. Under long-day light condition in *Arabidopsis*, molecular genetics has revealed that *CONSTANS* (*CO*), a transcription factor with two B-box-type zinc fingers, plays a central role in the photoperiod pathway, and its expression is controlled by both the circadian clock and light signals (Suárez-López *et al.* 2001; Böhlenius *et al.* 2006). And *CO* functions as a transcription factor that promotes flowering by inducing the expression of the florigen gene (*FT*) (Kardailsky *et al.* 1999; Corbesier *et al.* 2007; Kinmonth-Schultz *et al.* 2016). *CO* is one of 16 *CO-LIKE* (*COL*) genes that have been identified in the *Arabidopsis* genome (Robson *et al.* 2001). However, it has been shown that *CO* function may not be conserved in other COL proteins which contain B-boxes that are closely related to those of *CO*. For instance, changes in the expression levels of *COL1* and *COL2* have little effect on flowering time, whereas overexpression of *COL1* can impact circadian rhythm (Ledger *et al.* 2001), fruit ripening and stress responses (Chen *et al.* 2012). *COL3* is a positive regulator of red light signalling and root growth (Datta *et al.* 2006), and overexpression of *COL9* functions as a floral repressor (Cheng and Wang 2005). In contrast, *COL5*, a diurnal and circadian-regulated member of the *COL* family of proteins, induces flowering time by increasing *FT* expression in short-day grown *Arabidopsis* (Hassidim *et al.* 2009).

The radish (*Raphanus* genus) is one of the most important and popular vegetable crops in the Brassicaceae family. It is closely related to *Arabidopsis* and shows considerable genetic homology (Donovan *et al.* 2006). Distinct from cultivated radish, the winter-annual wild radish (*R. sativus* var. *raphanistroides*) is a principal vernalization-responsive plant (Sung and Amasino 2005) that grows spontaneously and widely along coastlines of Japan. There is close relationship between wild radish and cultivars (Yamagishi 2004; Yamane *et al.* 2009; Wang *et al.* 2015), indicating that increased understanding of the flowering characteristics of wild radish should contribute to the manipulation of crop environments to promote synchronous and effective flowering in cultivated production. Wild radish plants in northern and southern Japan exhibit obligate and facultative vernalization, respectively (Han *et al.* 2016). Specifically, northern populations require obligate vernalization for flowering, whereas southern populations display facultative vernalization, i.e., can flower without cold exposure. However, vernalization hastens the flowering of these ecotypes, thus offering an excellent system for genetic studies of flowering-time variations. Various studies have demonstrated the gene network mechanism that controls flowering time in winter-annual and summer-annual ecotypes of *A. thaliana* (Johanson *et al.* 2000; Michaels *et al.* 2003; Michaels *et al.* 2005; Vidigal *et al.* 2016; Lee *et al.* 2019). However, the genetic mechanisms underlying flowering-time variation in plants with obligate and facultative vernalization requirements, and the possible floral pathways and molecular mechanisms related to adaptive variations remain less clear.

In the current study, we investigated the expression patterns of genes that control flowering time in Japanese wild radish with different vernalization requirements (obligate and facultative) and identified the climatic cues that may act as natural selective forces shaping genetic variation in floral initiation. Specifically, we analysed of variations in flowering time in response to different cold and day-length treatments in accessions collected from the Hokkaido (northern lineage) and Okinawa (southern lineage) islands with contrasting climatic conditions. Understanding the integration of vernalization and photoperiod is important for clarifying how plants alter flowering time in response to various environmental signals. This work should improve our knowledge of how plants regulate flowering in response to multiple environmental cues.

## Materials and Methods

### Measurement of flowering time

**Figure 1.**
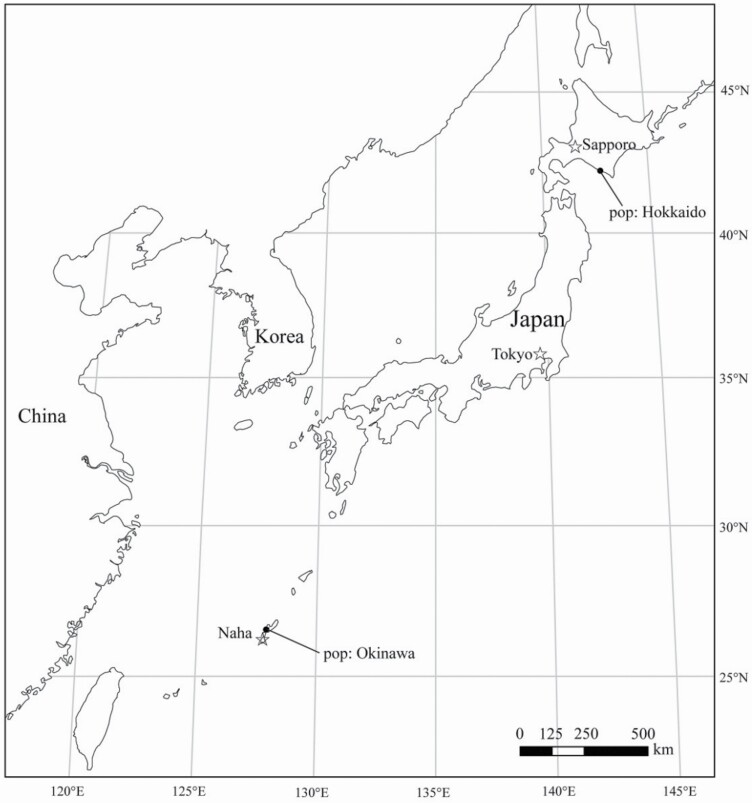
Sampling locations of Japanese wild radish populations.

To document flowering time in wild radish, ripe seeds were collected from Hokkaido Island and Okinawa Island (Fig. 1) as representative lineages. The genetic structure and north–south lineage of the two populations were proven by molecular genetics, as described in our previous paper (Han *et al.* 2016). To avoid the influence of genetic variation among individuals on gene expression, we collected seed samples from different closely spaced plants and then pooled eight individuals per site as a replicate. The effects of cold exposure (with and without) and day length (long- and short-day) on flowering induction were evaluated by using a 2 × 2 factorial experiment. The seeds were sown in Petri dishes containing filter paper soaked in tap water under darkness conditions at 21 °C for 3 days. The seedlings were then transplanted into Jiffy pots and placed within a growth chamber (21 °C, 10-h light (3000 lx)/14-h dark) for vegetative development for 1 week.

In the vernalized group, 1-week-old seedlings received cold treatment at 5 °C (10-h light/14-h dark) for 3 weeks. After vernalization, the plants were transplanted into plastic pots (diameter 9 cm × height 20 cm), then moved to an air-conditioned greenhouse (21 °C) in Kyoto, Japan (35°01′N/135°46′E) and subjected to natural short- or long-day light conditions. Under each experimental treatment of day length, 1-week-old seedlings without cold exposure were simultaneously grown in the same greenhouse as vernalized group. The short-day light treatment was from October to January of the following year, and long-day light treatment was from April to July. To reduce the effects of position, the pots were moved randomly every week. Flowering time was measured as the number of days from greenhouse planting until the appearance of the first flower. Based on our observations, the growing period for most samples ended ~100 days after greenhouse planting. Hence, the experiment was terminated at 120 days.

The flowering data were imported into SPSS v26.0. To compare the different groups and treatments, one-way analysis of variance (ANOVA) and LSD tests (least significant difference tests) were performed. The level of significance was set to 5 %. Additionally, Student’s *t-*test was used to determine significant differences between the long- and short-day length treatments, as well as between the vernalized and non-vernalized treatments. We also investigated the major climatic factors that may act as natural selective forces shaping variation in flowering time, including temperature and photoperiod. Climatic data from 1981 to 2010 were obtained from the Japan Meteorological Agency (JMA) located on Hokkaido and Okinawa islands, according to the World Meteorological Organization (WMO) Technical Regulations. Additionally, the mean day length per month in Hokkaido, Okinawa and Kyoto (location of cultivation experiment) was collected from weather station (https://weather-stats.com/japan). These data are presented in **Supporting Information—Table S1****and****Fig. S3**.

### Flowering-time gene expression analyses

To better understand the roles of cold exposure and photoperiod in determining flowering time in wild radish, we investigated the expression levels of major flowering-time genes, including *RsFLC*, *RsCOL5* (*A*&*B*) and *RsFT*. The functions of the studied *RsFLC*, *RsCOL5* (*A*&*B*) and *RsFT* genes are conserved between *Arabidopsis* and wild radish (Yi *et al.* 2014; Kitashiba and Yokoi 2017; Hu *et al.* 2018), and play key roles in the vernalization pathway, photoperiod pathway and flowering pathway integration, respectively (Putterill *et al.* 2004; Nie *et al.* 2016). We aligned four homologs of the *COL5* gene (RSG21446.t1, RSG35888.t1, RSG44241.t1 and RSG42288.t1) in wild radish to AT5G57660.1 in *Arabidopsis* and the identity were 79.47, 79.42, 15.64 and 14.98 %, respectively. We eventually selected two genes with higher alignment, RSG35888.t1 (*RsCOL5-A*) and RSG21446.t1 (*RsCOL5-B*), as indicators of *CO-LIKE* 5. **Supporting Information—Table S2** showed the corresponding homologs of *FLC*, *COL5* (*A*&*B*) and *FT* in *R. sativus* (https://www.ncbi.nlm.nih.gov/assembly/GCA_001047155.1) and *A. thaliana* (http://www.arabidopsis.org/).

The seeds collected from Hokkaido and Okinawa islands were sown in Petri dishes containing filter paper soaked in tap water under darkness at 21 °C for 3 days. Afterwards, the seedlings of vernalized groups were transplanted into plastic pots and placed inside a growth chamber for vernalization (5 °C, 16-h light/8-h dark) for 4 weeks. At the end of cold treatment, the non-vernalized and vernalized groups were moved together into the same growth chamber at 21 °C, 16-h light/8-h dark. Fresh leaves proximal to the shoot apex were collected from three individuals every 4 h after the lights were turned on (ZT0, Zeitgeber time 0) at the 11-day and 22-day growth stages. Herein, ZT represents a standardized 24-h notation of the phase in an entrained circadian cycle in which ZT0 indicates the beginning of the day or light phase and ZT16 (16 h after light on) refers to the time at which the light-to-dark transition occurs. The 11-day/22-day of growth stages were recorded from the days for planting in the growth chamber for not vernalized group/the end of the cold treatment for vernalized group.

As floral induction genes exhibit the highest expression before bolting (Wang *et al.* 2018), we only harvested the vernalized southern group at the 11-day growth stage (just before bolting), while the other three groups (i.e. non-vernalized southern, vernalized northern and non-vernalized northern) were analysed at the 11-day and 22-day growth stages. Immediately after harvesting, the samples were flash-frozen in liquid nitrogen. RNA was extracted using the RNeasy plant mini kit (Qiagen GmbH, Hilden, Germany) in combination with DNase treatment using the RNase-free DNase Set (Qiagen GmbH) according to the manufacturer’s instructions. In total, 1 µg of RNA was used for cDNA synthesis using the QuantiTect Reverse Transcription Kit (Qiagen GmbH).

Quantitative real-time polymerase chain reaction (qRT–PCR) was performed on a StepOne™ Real-Time PCR System (Applied Biosystems, Foster City, CA, USA), using SYBR Premix Ex Taq II (including *Taq* DNA polymerase, reaction buffer and deoxynucleotide triphosphate mixture) (Takara Bio Inc.). Each reaction was carried out in a total volume of 20 μL, consisting of 10 μL of SYBR Premix, 2 μL of cDNA sample (diluted 1:3) and 0.4 μM of the forward and reverse primers. qRT–PCR was performed at 95 °C for 30 s followed by 40 cycles of 95 °C for 10 s, 58 °C for 30 s and 72 °C for 1 min and a melting curve program of 95 °C for 15 s, 60 °C for 1 min and 95 °C for 15 s. Constitutively expressed *Rs-UBQ* was used as a reference gene for normalization (Ayarpadikannan *et al.* 2014). Gene expression was quantified using the ΔΔCt method (Livak and Schmittgen 2001).

## Results

### Variation in flowering time

**Figure 2.**
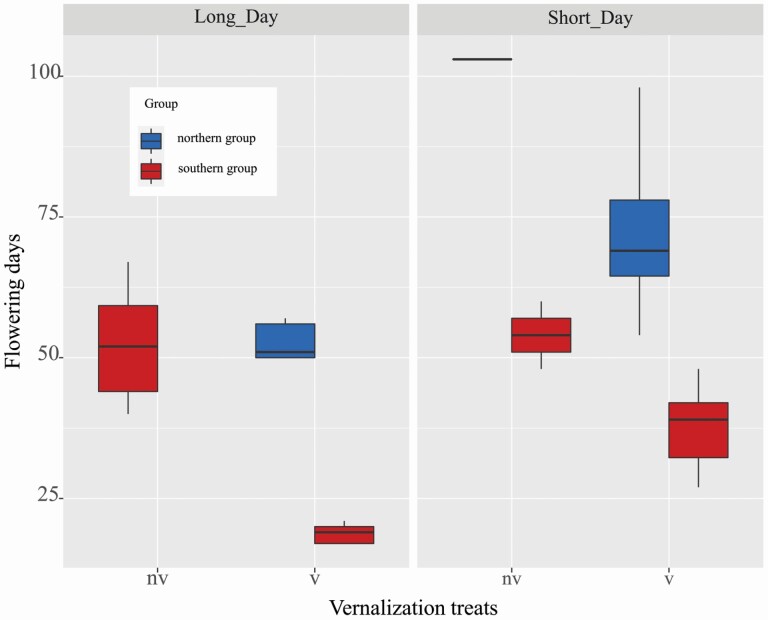
Flowering-time variations observed in southern and northern accessions. nv: non-vernalized; v: vernalized. Not-flowered non-vernalized northern groups under long-day and short-day conditions were excluded in this figure. Each box represented the interquartile range which contains 50 % of the values and the median (horizontal line across the box); statistical analysis of data was carried out by one-way ANOVA followed by the LSD test. Different letters in each group indicate significant differences at *P* < 0.05.

To verify different vernalization requirements (obligate and facultative), we investigated variations in the flowering time of wild radish from Hokkaido and Okinawa islands under long- and short-day light conditions (Fig. 2). Student’s *t*-test results showed significant differences in flowering time between the vernalized and non-vernalized treatments (*P* < 0.05). With respect to the northern accessions, under long-day photoperiod treatment with vernalization, flowering onset ranged from 40 to 92 days (mean, 54.3; median, 51); without vernalization, however, regardless of long- or short-day light conditions, flowering failed to occur by 120 days after planting **[see****Supporting Information—Fig. S1****]**. In contrast, the vernalized southern accessions flowered significantly earlier (17–21 days; mean, 18.6; median, 19) than the non-vernalized samples (40–67 days; mean, 52.3; median, 52).

Interestingly, the southern lineage was able to flower without cold exposure. Significant differences in flowering time were found between northern and southern groups within the same treatment. Patterns of responses between short- and long-day light treatment were similar, but not statistically significance (*P* = 0.277 > 0.1) (Fig. 2). Additionally, we found an increasing linear relationship between the number of leaves and number of days from planting to flowering **[see****Supporting Information—Fig. S2****]**. Linear fitting analysis showed that *R. sativus* exhibited multi-leaves with late flowering and sparse leaves with early flowering. The positive correlation indicated the north–south accessions were in the roughly consistent growth rates in vegetative phase until they flowered. Taken together, the southern accessions displayed facultative vernalization requirements while northern accessions were obligate type, consistent with previous study (Han *et al.* 2016).

To explore the environmental cues that may act as natural selective forces shaping variation in flowering time, we analysed major climatic factors, such as temperature and photoperiod, on the Hokkaido and Okinawa islands **[see****Supporting Information—Table S1****and****Fig. S3****]**. Mean temperature, daily maximum temperature, daily minimum temperature sunshine duration and day length differed greatly between the northern and southern regions. Field surveys conducted under natural conditions indicated that the southern population germinated in the fall and flowering was delayed until the following spring, whereas the northern population received vernalization of imbibed seed in winter, germinated in spring and flowered in the summer of the same year. These different life cycles may allow wild radish to adapt to seasonal and environmental variations.

### Expressions levels of flowering-time-related genes

To test the effect of photoperiod on flora induction, we separated all the treatments into two groups (with long-day or short-day light condition) and performed the Student’s *t*-test. Results showed no significant differences in flowering time between the two groups (Fig. 2). In addition, considering *R. sativus* is a facultative long-day light plant, we investigated the responses of gene expression to long-day light (closest to natural conditions) with vernalized and non-vernalized treatments to explore the associations of *RsFLC* and *RsCOL5* (*A*&*B*) expression and flowering regulation in Japanese wild radish.

**Figure 3.**
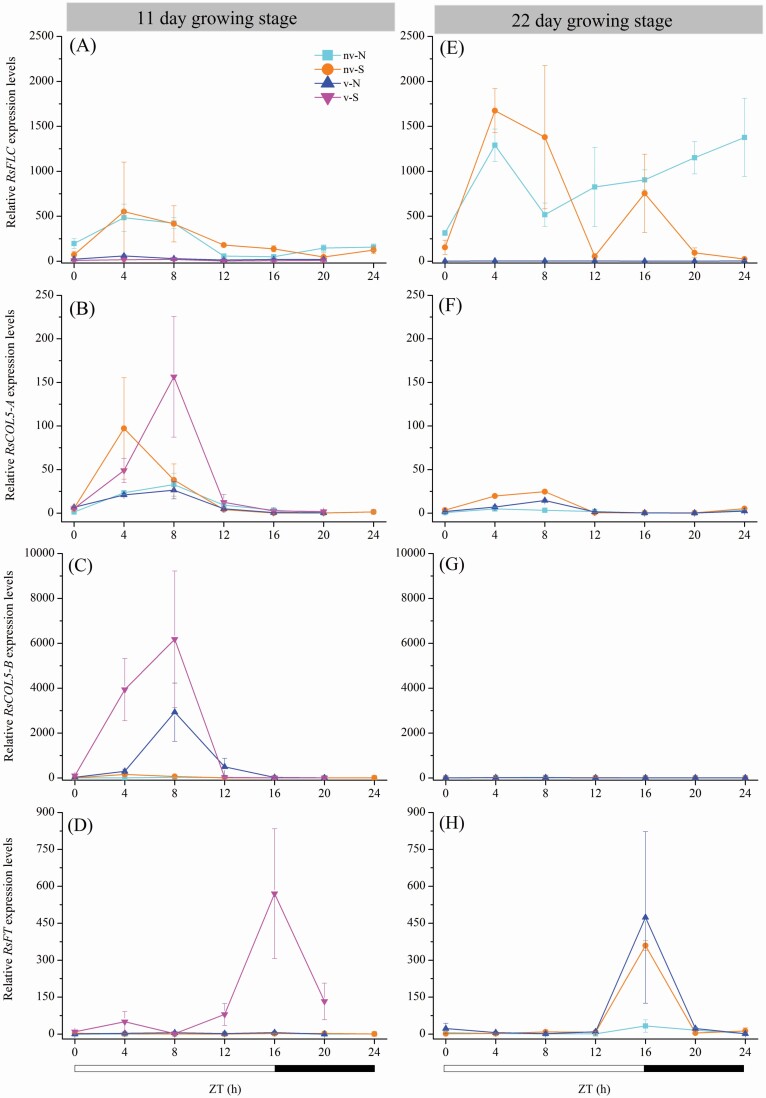
Relative expression levels of *RsFLC*, *RsCOL5-A*, *RsCOL5-B* and *RsFT* in northern and southern accessions of wild radish at the 11-day (left: A–D) and 22-day (right: E–H) growth stages. Black and white bars at the bottom indicate light (16 h) and dark (8 h) phases, respectively (ZT, Zeitgeber time). The error bars represent standard errors of three biological replicates, which are the mean values of three technical replicates.

In the vernalized southern accessions, the expression of *RsFLC* was reduced after vernalization and was almost undetectable (Fig. 3A). In contrast, the expression levels of *RsCOL5-A* and *RsCOL5-B* increased from ZT4, peaked at ZT8 and then decreased to a near-zero level (Fig. 3B and C). *RsFT* was strongly activated at the end of the photoperiod (ZT16) but was repressed at other times of the day (Fig. 3D). Notably, the kinetic expression of *RsFT* was possibly related to the abundance of *RsCOL5-A* and *RsCOL5-B*. Furthermore, the expression of *RsCOL5* (*A*&*B*) mRNA may result in an abundance of *RsFT*, a key flowering-time integrator, which contributed to the early onset of flowering. Thereby, genes known to mediate the flowering response to photoperiod in *A. thaliana* may be also regulated the flowering-time response to vernalization in wild radish.

In the non-vernalized southern accessions, both the abundance and transcriptional activity of *RsFLC* were high throughout the experiment (Fig. 3A and E). *FLC* delays flowering by repressing the expression of *FT* genes involved in floral induction (Searle *et al.* 2006; Turck *et al.* 2008). Thus, one could speculate that the observed low expression of *RsFT* was probably generated by the repression of *RsFLC* (Fig. 3D). In addition, *RsCOL5-B* remained downregulated through all stages in the non-vernalized southern accessions. In contrast, the expression of *RsCOL5-A* was strongly detected at the 11-day growth stage. Furthermore, *RsFT* expression was high at the 22-day growth stage and peaked at ZT16, accompanied by high *RsFLC* transcript levels. The high expression of *RsFT* at 22 days maybe due to the presence of *RsCOL5-A* activity. It is possible that *RsCOL5-A* alone was not sufficient to induce flowering when *RsFLC* was highly expressed at the 11-day growth stage.

In the vernalized northern accessions, the *RsFLC* mRNA levels were greatly reduced by cold exposure (Fig. 3), in agreement with the findings of Alexandre and Hennig (2008). During the experimental period, *RsCOL5-A* was expressed at a low level at most time points. In contrast, at the 11-day growth stage, *RsCOL5-B* increased dramatically from ZT4 and peaked at ZT8. It is assumed that flowering is promoted when plants pass through an accumulation phase to reach a sufficient *FT* expression level. Interestingly, the peaks in *RsFT* expression in the vernalized northern population at 22-day overlapped perfectly with the vernalized southern population at 11-day. As overexpression of *COL5* can induce *FT* in *Arabidopsis* (Hassidim *et al.* 2009), the high *RsCOL5-B* expression, rather than *RsCOL5-A* expression, may be correlated with the elevation in *RsFT* mRNA levels. Flowering in the vernalized northern accessions was plausibly influenced by an increase in the expression of floral promoter *RsCOL5-B* at the 11-day growth stage and a reduction in the expression of floral repressor *RsFLC*.

In the non-vernalized northern accessions, *RsFLC* was highly expressed at the 22-day growth stage (Fig. 3E) and generally exhibited broad peaks at ZT4. Expression levels continued to increase from ZT8, reaching another peak at the end of the dark period (ZT24) at 22 days. Both *RsCOL5-A* and *RsCOL5-B* were poorly expressed at the 11-day and 22-day growth stages. As the plants grew, *RsFT* was nearly completely repressed and was undetectable at most time periods, consistent with a potential mechanism in which is strongly suppressed by *RsFLC* and correlated with *RsCOL5-A* and *RsCOL5-B* reduction. Interestingly, in this way, *RsFLC* would be repressing *RsFT* expression and thus preventing flowering in the non-vernalized northern accessions. These observations provided hints that the increased *RsFLC* and the downregulation of *RsCOL5-A* and *RsCOL5-B* collectively assisted in the maintenance of a stable downregulated of the floral transition of the *RsFT* gene, ultimately resulting in vegetative growth without flowering.

## Discussion

### Differential vernalization requirements of wild radish

Flowering time is sensitive to climatic signals, including prolonged cold (vernalization) and day length (photoperiod), which serves as ecological cues to ensure that reproductive effort occurs under optimal seasonal environments. Thus, natural variation in flowering may reflect local adaptation to environmental cues. In this research, we investigated natural variation in flowering time of wild radish and the role of two major climatic cues (cold exposure and day length) in regulating flowering transition. In the cultivation experiment, almost the entire northern population remained in the vegetative stage throughout the 120-day growth period, with no signs of floral transition unless exposed to prolonged cold. Conversely, flowering took place in the southern accessions regardless of cold/light treatment, but vernalization significantly promoted flowering time. The southern lineage flowered significantly earlier under cold exposure, but still flowered without vernalization. Thus, vernalization was not required for flowering in the southern group, although it substantially shortened the timing of flowering. These observations confirmed the obligate and facultative vernalization requirements of wild radish, consistent with our previous study (Han *et al.* 2016). The different requirements and responses to vernalization between the northern and southern populations may reflect the action of different selective pressures shaped by their local habitats.

### Correlation between RsFLC and RsCOL5 (A&B) expression patterns

Genetic analysis of *A. thaliana* has identified numerous pathways that control the timing of floral transition (Irish 2010). Photoperiod is a major seasonal cue for flowering in *A. thaliana* and studies have demonstrated that vernalization can overcome the obstruction of photoperiod floral induction (Engelmann and Purugganan 2006). These two flowering pathways (i.e. vernalization and photoperiod) initiate various floral pathway integrator genes, e.g. *FT* and *SOC1* (*Suppressor of overexpression of CO1*), to trigger floral transition (Song *et al.* 2015). The major role of *FLC* in the vernalization pathway is to repress flowering by inhibiting the expression of *FT* and *SOC1*. Vernalization leads to reduced *FLC* mRNA and protein levels, thereby removing the *FLC*-mediated repression of flowering. Hence, exposure to prolonged periods of cold is opposite to the function of the long-day light floral promoter, *CO*, which activates floral pathway integrator genes.

The molecular basis of the opposing effects of *FLC* and *CO* (i.e. vernalization and photoperiod pathways) has received increasing attention (Hepworth *et al.* 2002; Ream *et al.* 2014). For instance, overexpression of *FLC* blocks the activation of *SOC1* by *CO* (Hepworth *et al.* 2002). In addition, high levels of *FLC* can cause negative regulation of *CRY2* (*Cryptochrome* 2), thereby preventing *CRY2* from promoting of *CO* expression (El-Assal *et al.* 2003). Consistently, we identified found opposite activity between *RsFLC* and *RsCOL5* (*A*&*B*) in the regulation of flowering time in the northern and southern accessions. In the northern accessions, flowering in the non-vernalized group was primarily affected by *RsFLC*: i.e. overexpression of *RsFLC* repressed floral initiation, whereas the absence of *RsFLC* allowed *RsCOL5-B* to promote flowering after vernalization. In the southern accessions, following the accumulation of *RsCOL5-A* over time, *RsFT* increased rapidly, and flowering was successful, despite the action of *RsFLC* as a floral ‘brake’. Thus, the promoting function of *RsCOL5-A* may have been stronger than the floral repressor function of *RsFLC* over time, resulting in sufficient expression of *RsFT*. Thus, cold exposure and high *RsCOL5-A* and *RsCOL5-B* expression may have collectively resulted in significantly earlier flowering times. In contrast, without cold exposure, flowering time was promoted by the overexpression of *RsCOL5-A*, despite high *RsFLC* transcript levels. However, the mechanism of *RsCOL5-A* in upregulating *RsFT* remains unclear, and further studies using mutants and overexpression lines are required. In addition, the expression patterns did not provide evidence for interactions among all genes; thus, further genetic studies and *in vivo* experiments are needed to verify protein–protein or protein–DNA interactions.

As the southern populations did not require vernalization, we studied the molecular mechanism underlying early flowering in these plants. Summer-annual strains of *Arabidopsis* consistently display low *FLC* expression to promote flowering, resulting from the absence of *FRI* activity (Johanson *et al.* 2000) or disruption of the *FLC* regulatory sequences by transposons (Michaels *et al.* 2003). In wild radish, however, high *RsFT* activity and the consistent early flowering phenotype implied that *RsCOL5-A* may initiate flowering in the presence of the floral repressor *RsFLC*. Many studies have reported that the vernalization pathway can override other floral pathways (Tadege *et al.* 2003; Alexandre and Hennig 2008; Miller *et al.* 2008). In our work, however, *RsCOL5-A* in the southern accessions induced flowering, even under a background of *RsFLC* expression, indicating a preference for the photoperiod pathway rather than the vernalization pathway. However, further studies are required to explore the specific molecular mechanisms underlying the dominance of the photoperiod pathway in non-vernalized southern populations of Japanese wild radish, i.e. the floral promotion of *RsCOL5-A* under high *RsFLC* expression. Such research will offer new hints on the molecular mechanisms underpinning adaptive variation in flowering time.

### Natural selection may affect floral pathway dominance

Wild radish is a genetically diverse and highly adaptable species that thrives in a wide range of environments (Madhou *et al.* 2005). The daily minimum temperature from October to February ranged from 14.6 to 23.1 °C in Okinawa (Naha), but from −7 to 7.5 °C in Hokkaido (Sapporo) **[see****Supporting Information—Table S1****]**. Considering that the optimum vernalization temperature ranges from 1 to 7 °C in most species (Amasino 2010; Duncan *et al.* 2015) and the daily minimum temperature in the southern region is higher than the favourable vernalization temperature, it is plausible that the southern accessions were unable to undergo vernalization. In contrast, the northern temperatures are cold enough for vernalization to initiate flowering in the northern populations. These observations confirm the different vernalization requirements of the northern and southern accessions (i.e. obligate and facultative, respectively) in agreement with earlier research (Han *et al.* 2016). Based on field investigation, the day length required for flowering was much shorter for the southern population than for the northern population under field conditions. Specifically, the southern population tended to favour short-day light condition (~11 h in February; **see****Supporting Information—Fig. S3**), whereas the northern accessions preferred long-day light condition (~14.5 h in May; **see****Supporting Information—Fig. S3**) after prolonged cold exposure to initiate flowering. These results imply that differences in dominance of floral pathways may be generated by natural selection.

Flowering time is an important determinant of fitness in a variable environment and responds plastically to seasonal cues involving temperature and photoperiod (Amasino 2010). Based on field surveys, we found that the adaptive strategies of Japanese wild radish were nearly consistent with the winter-summer annual ecotypes of *A. thaliana* covering a range of latitudes (Engelmann and Purugganan 2006; Vidigal *et al.* 2016). Specifically, the southern accessions, like the winter-annual ecotype of *A. thaliana*, germinated in the autumn and overwintered as rosettes, where they experienced short-day lengths, and then flowered in the following spring. In contrast, northern samples germinated in spring and flowered and set seed in the same summer or autumn season, consistent with the summer-annual ecotype of *A. thaliana.* However, unlike the winter-annual *Arabidopsis* ecotype, the southern lineage of Japanese wild radish did not require vernalization to trigger flowering. In contrast, although the northern wild radish samples resembled summer-annual of *Arabidopsis*, they needed vernalization to promote floral transition. The different life-history traits control the time invested in vegetative growth and time to reproduction, reflecting adaptations of the life cycle to the broad environmental and ecological diversity.

## Conclusions

Flowering time is a developmental transition that is sensitive to ecological cues and can adapt to seasonal and environmental variations. Our research showed that the *RsFLC* and *RsCOL5* (*A*&*B*) genes interact to regulate flowering time and floral pathway dominance is affected by their seasonal cues. Notably, the northern accessions may modulate flowering time primarily through the vernalization pathway, whereas the southern accessions may preferentially mediate flowering time based on the photoperiod pathway under non-vernalization conditions. Thus, these mechanisms help initiate optimal flowering time in natural populations in response to local climatic cues. Our results indicate rich natural diversity in photoperiod and vernalization requirements to exploit for understanding gene networks controlling flowering, which will offer new hints about the molecular mechanism underpinning adaptive variation of flowering time, adapting to ongoing and future climate change. However, further studies are required to understand how plants alter flowering pathway activity to adapt to growth in different geographical locations and how plants balance the effects of different environmental stimuli on flowering time at a more precise molecular level, which is important for the efficient manipulation of flowering in crop production.

## Supporting Information

The following additional information is available in the online version of this article—

**Table S1.** The monthly/annual climate normal for major observatories in Sapporo and Naha in Japan, calculated from 1981 to 2010.

**Table S2.** Locus information and list of the primers for gene expression analyses.

**Figure S1.** Vegetative growing and flowering ones at 60-day (A) and 120-day (B) after the wild radish was planted in the greenhouse. In the respective picture, it displayed not-flowering (left) of non-vernalized northern individual and flowering (right) individuals from the southern region.

**Figure S2.** Leave number versus flowering time and its linear fitting approach.

**Figure S3.** Mean day length per month of the studied locations. Day length: the number of hours when sun is above the horizon line.

plab039_suppl_Supplementary_Materials

## Data Availability

Data and gene sequences used in this paper are available on Figshare: https://figshare.com/s/4be0a586c85899a7e5a3.

## Notes

**Phenome, Genome & Environment. Chief Editor:** Colleen Doherty
