# Supplementary material for: Association between RsFT, RsFLC and RsCOL5 (A&B) expression and flowering regulation in Japanese wild radish
Source: AoB Plants. 2021 Jun 23;13(4):plab039. doi: 10.1093/aobpla/plab039 (PMC8286712; doi:10.1093/aobpla/plab039)
Supplement: plab039_suppl_Supplementary_Materials [file plab039_suppl_supplementary_materials.pdf]

Supporting information

Table S1 The monthly/annual climate normal for major observatories in Sapporo and Naha in Japan, calculated from 1981 to 2010

Month	Mean temperature (℃)		Daily Maximum temperature (℃)		Daily Minimum temperature (℃)		Sunshine duration (h)	
	Sapporo	Naha	Sapporo	Naha	Sapporo	Naha	Sapporo	Naha
Jan.	-3.6	17	-0.6	19.5	-7	14.6	92.5	94.2
Feb.	-3.1	17.1	0.1	19.8	-6.6	14.8	104	87.1
Mar.	0.6	18.9	4	21.7	-2.9	16.5	146.6	108.3
Apr.	7.1	21.4	11.5	24.1	3.2	19	176.5	123.8
May	12.4	24	17.3	26.7	8.3	21.8	198.4	145.8
Jun.	16.7	26.8	21.5	29.4	12.9	24.8	187.8	163.6
Jul.	20.5	28.9	24.9	31.8	17.3	26.8	164.9	238.8
Aug.	22.3	28.7	26.4	31.5	19.1	26.6	171	215
Sep	18.1	27.6	22.4	30.4	14.2	25.5	160.5	188.9
Oct.	11.8	25.2	16.2	27.9	7.5	23.1	152.3	169.6
Nov.	4.9	22.1	8.5	24.6	1.3	19.9	100	123
Dec.	-0.9	18.7	2.1	21.2	-4.1	16.3	85.9	115.6
Annual	8.9	23.1	12.9	25.7	5.3	20.8	1740.4	1774

Table S2 Locus information and list of the primers for gene expression analyses

Locus	Primer Sequences (5′-3′)	Radish gene ID	TAIR10 Top Hit
RsFLC	F: TGAGACTGCCCTATCTCTAACT<br>R: CGGATATTTGTGCACGTGACA	RSG13912.t1	AT5G10140.1
RsCOL5-A	F: GGAGGCTAGGGTTTTGAGGT<br>R: ACACCGTAATGTCCAGCTGA	RSG35888.t1	AT5G57660.1
RsCOL5-B	F: GAACACCACAGGCGATCAAG<br>R: GAACCGGCCTTTGATCCTG	RSG21446.t1	AT5G57660.1
RsFT	F: CGTATCGTGCTGGTATTGTTCCG<br>R: ACTTCTTCGTCCTCCACAGCCA	RSG32193.t1	AT1G65480.1
RsUbiquitin	F: TCATCCGACACCATCGACAATG<br>R: ATGACGATCGAAATGACTCGCC	RSG21492.t1	AT2G47110.1

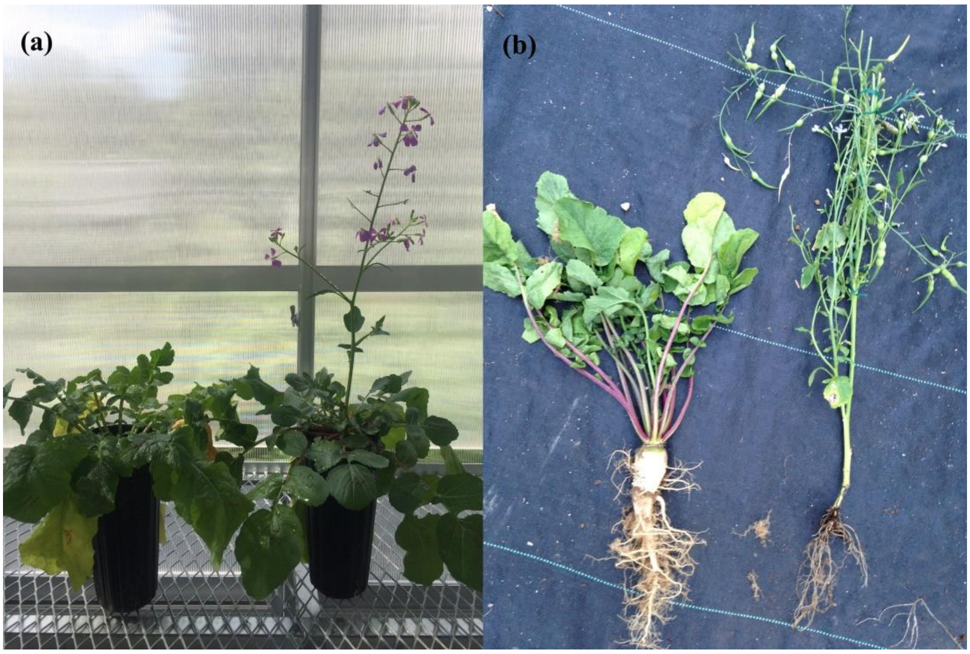

Fig. S1 Vegetative growing and flowering ones at 60-day (a) and 120-day (b) after the wild radish was planted in the greenhouse. In the respective picture, it displayed not-flowering (left) of non-vernalized northern individual and flowering (right) individuals from the southern region.

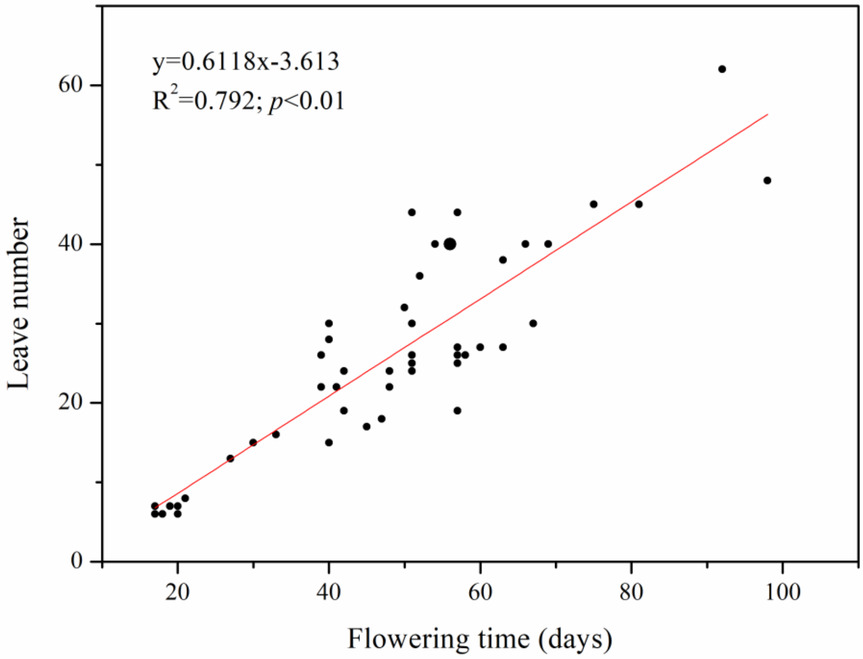

Fig. S2 Leave number versus Flowering time and its linear fitting approach.

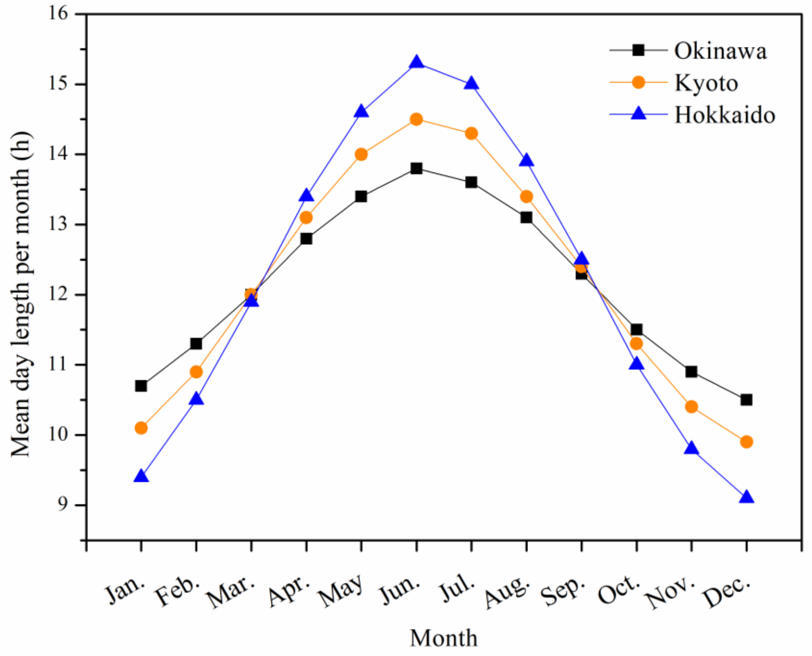

Fig. S3 Mean day length per month of the studied locations.

Day length: the number of hours when sun is above the horizon line.
